# The Profiling of 179 miRNA Expression in Serum from Limb Girdle Muscular Dystrophy Patients and Healthy Controls

**DOI:** 10.3390/ijms242417402

**Published:** 2023-12-12

**Authors:** Francesca Magri, Laura Napoli, Michela Ripolone, Patrizia Ciscato, Maurizio Moggio, Stefania Corti, Giacomo Pietro Comi, Monica Sciacco, Simona Zanotti

**Affiliations:** 1 Neurology Unit, Fondazione IRCCS Ca’ Granda Ospedale Maggiore Policlinico, 20122 Milan, Italy; 2 Neuromuscular and Rare Disease Unit, Fondazione IRCCS Ca’ Granda Ospedale Maggiore Policlinico, 20122 Milan, Italymaurizio.moggio@policlinico.mi.it (M.M.);; 3 Dino Ferrari Centre, Department of Pathophysiology and Transplantation (DEPT), University of Milan, 20122 Milan, Italy

**Keywords:** limb girdle muscle dystrophy, miRNAs, fibrosis, inflammation, atrophy, biomarkers

## Abstract

Limb girdle muscular dystrophies (LGMDs) are a group of genetically inherited neuromuscular diseases with a very variable clinical presentation and overlapping traits. Over the last few years there has been an increasing interest in the use of non-invasive circulating biomarkers to monitor disease progression and to evaluate the efficacy of therapeutic approaches. Our aim was to identify the miRNA signature with potential value for LGMD patient screening and stratification. Using miRCURY LNA miRNA qPCR Serum/Plasma Panel, we analyzed 179 miRNAs from 16 patients, divided in four pools based on their genetic diagnosis, and from healthy controls. The miRNAs analysis showed a total of 107 dysregulated miRNAs in LGMD patients when compared to the healthy controls. After filtering via skeletal tissue expression and gene/pathways target analysis, the number of dysregulated miRNAs drastically reduced. Six selected miRNAs—let-7f-5p (in LGMDR1), miR-20a-5p (in LGMDR2), miR-130b-5p, miR-378a-5p (both in LGMDR3), miR-376c-3p and miR-382-5p (both in LGMDR4)—whose expression was significantly lower compared to controls in the different LGMD pools, were further investigated. The bioinformatic analysis of the target genes in each selected miRNA revealed ECM–receptor interaction and TGF-beta signaling as the most involved pathways. The correlation analysis showed a good correlation of let-7f-5p with fibrosis and with the cross sectional area of type I and type II fibers, while miR-130b-5p showed a good correlation with the age of onset of the disease. The receiver operating characteristic curves showed how single miRNAs were able to discriminate a specific group of LGMD patients and how the combination of six miRNAs was able to discriminate LGMD patients from controls.

## 1. Introduction

Limb girdle muscular dystrophies (LGMDs) are a group of genetically inherited neuromuscular diseases. The most recent classification encompasses 31 genetically transmitted LGMD variants including autosomal dominant, autosomal recessive and X-linked forms. The most common are calpainopathies, dysferlinopathies, sarcoglycanopathies, dystroglycanopathies and anoctaminopathies [1]. The clinical presentation is quite variable in accordance with each disorder’s main features, i.e., the groups of primarily affected muscles, the degree of weakness, and the age of onset and progression rate. Though the diagnostic process is well defined for most of these pathologies, some aspects, including differences in the age of onset or in the disease progression, are still poorly defined.

Calpainopathy (LGMDR1) is caused by pathogenic variants in the *CAPN3* gene codifying for a non-lysosomal calcium-dependent cysteine protease. The primary symptom is a progressive worsening of muscle weakness of the hip and shoulder muscles, enlarged calf muscles, shortening and hardening of muscles leading to contractures, scoliosis, and the winging of the shoulder blades. Dysferlinopathy (LGMDR2), caused by pathogenic variants in the *DYSF* gene, is characterized by weakness and atrophy of the pelvic and shoulder girdle muscles that begin in adolescence or young adulthood and have a slow progression [2]. Sarcoglycanopathies (LGMDR3-6) are caused by pathogenic variants in one of *SGCA*, *SGCB*, *SGCG* and *SGCD* genes, encoding for sarcoglycans. The clinical phenotype of sarcoglycanopathies ranges from a severe Duchenne-like muscular dystrophy to forms with mild muscular involvement. The most frequent clinical phenotype includes progressive muscle weakness and atrophy, predominantly of the shoulder and pelvic girdles; respiratory and cardiac involvement is often present. This wide genotype–phenotype variability further complicates disease progression predictability. 

Even if LGMDs are genetically determined, skeletal muscle analysis—by both immunohistochemistry and Western blot—is frequently still mandatory to allow the correct diagnostic process. Muscle biopsy, however, is invasive and expensive, and is not suitable to evaluate the efficacy of a therapeutic intervention or to follow the disease progression.

Therefore, over the last few years, there has been an increasing interest in identifying non-invasive biomarkers to both monitor disease progression and evaluate the efficacy of therapeutic approaches. Indeed, to date, no efficient therapies are available for LGMD patients, even if some therapies are being developed and several clinical trials are on-going [1].

The most common non-invasive biomarker in muscular dystrophy is the serum creatine kinase (CK) level. However, it is influenced by several external conditions (cramps, physical activity, age and ethnicity) and, therefore, is not so reliable for disease assessment and progression or for evaluating the efficacy of therapeutic intervention. CK variations are also rather unspecific, with elevated values in plasma being detected also in other myopathies, in neurogenic disorders, and in cases of muscle trauma or extreme effort. More recently, the serum isoform of troponin I, TNNI-2, expressed in fast skeletal muscle fibers, has been used as a biomarker in Duchenne Muscle Dystrophy (DMD) patients and has been found to be increased along with serum CK levels, implicating the early involvement of fast skeletal muscle fibers in dystrophinopathies [3]. Regarding DMD patients, other circulating proteins are being investigated as possible biomarkers, namely matrix metalloproteinase-9 and the tissue inhibitors of metalloproteinase-1, both found increased in DMD patient sera [4]. In LGMDR2, proteins such as myosin light chain 3 (MYL3), fatty acid binding protein 3 (FABP3) [5], and myofibrillar structural protein myomesin-3 (MYOM3) were investigated as circulating biomarkers [6].

MicroRNAs (miRNAs), short endogenous RNAs which regulate the silencing of the expression of target genes post-transcriptionally, stand out as intriguing and promising circulating biomarkers, also in consideration of their relative stability in tissues and biofluids (plasma, serum, urine and saliva). An altered specific miRNA profile was identified in neurodegenerative diseases [7,8,9], in cancers (oncomiRs) [10,11], and in several other pathological conditions such as inflammation [12], cardiac fibrosis [13] and skeletal muscle fibrosis [14,15].

In muscle biology, miRNAs’ network plays an important role during skeletal muscle development and regeneration [16]. In skeletal muscle biopsies, Eisenberg et al., (2007) demonstrated a distinct miRNA profile in ten different primary muscular disorders [17]. Subsequently, numerous studies allowed for the identification of a restrictive group of miRNAs highly expressed in muscle tissue, the myomiRs (miR-1, miR-133a, miR-133b, miR-206, miR-208a, miR-208b, mir-499a and miR-499b), whose expression levels were correlated with the disease severity and clinical assessments of the patients [18,19,20,21]. In DMD patients, myomiRs were found to be increased in the sera of patients and mdx mice [19] even if a correlation between myomiRs’ expression levels and disease progression was not clear.

The purpose of our study is to identify specific serum miRNA profiles, excluding myomiRs, in a cohort of LGMD patients using a serum miRNA-focused PCR Panel containing 179 different circulating miRNAs. The identification of LGMD-specific miRNA profiles would contribute to LGMD patient stratification and to a better targeting of potential future therapeutic approaches. Furthermore, the identification of specific altered miRNAs in LGMD patients’ sera could provide useful non-invasive biomarkers to monitor disease progression and the efficacy of potential pharmacological treatments.

## 2. Results

### 2.1. Clinical Aspects and Muscle Biopsy

All patients involved had been genetically diagnosed and were symptomatic at the time of data collection. Table 1 shows the demographic, clinical and functional features of the patients. For each LGMD pathological group, four patients were recruited as reported in Table 1.

The mean age of the patient was 33.70 ± 15.00 years, with a lower age in the LGMDR4 group (19.50 ± 6.61 years), and the mean follow-up period after the disease onset was 18.85 ± 12.40 years. The LGMDR4 patient showed an earlier onset and a faster disease progression. A total of 5/16 (31.2%) patients had lost independent ambulation at the time of evaluation. Cardiac involvement was present in 4/16 (25%) patients and a respiratory restrictive pattern requiring non-invasive ventilation was detected in 3/16 (18.7%) patients. Serum CK levels were significantly increased in all patients. At functional evaluations, all patients showed impairment, especially in the items regarding proximal and axial strength, while distal functions and upper limb strength, evaluated through motor function measurement (MFM) dimension 3 and the performance upper limb (PUL) scale, were relatively preserved. Low scores were recorded by use of a North Star assessment for limb girdle (NSAD), especially in LGMDR1 patients. The six-minute walking (6MWT) performance was markedly deficient in LGMDR1, LGMDR2 and LGMDR3 patients compared to normal values and only slightly reduced in the two ambulant LGMDR4 patients (Table 1) [22]. The overall functional performances were worse in the LGMDR4 group.

**Figure 1:**
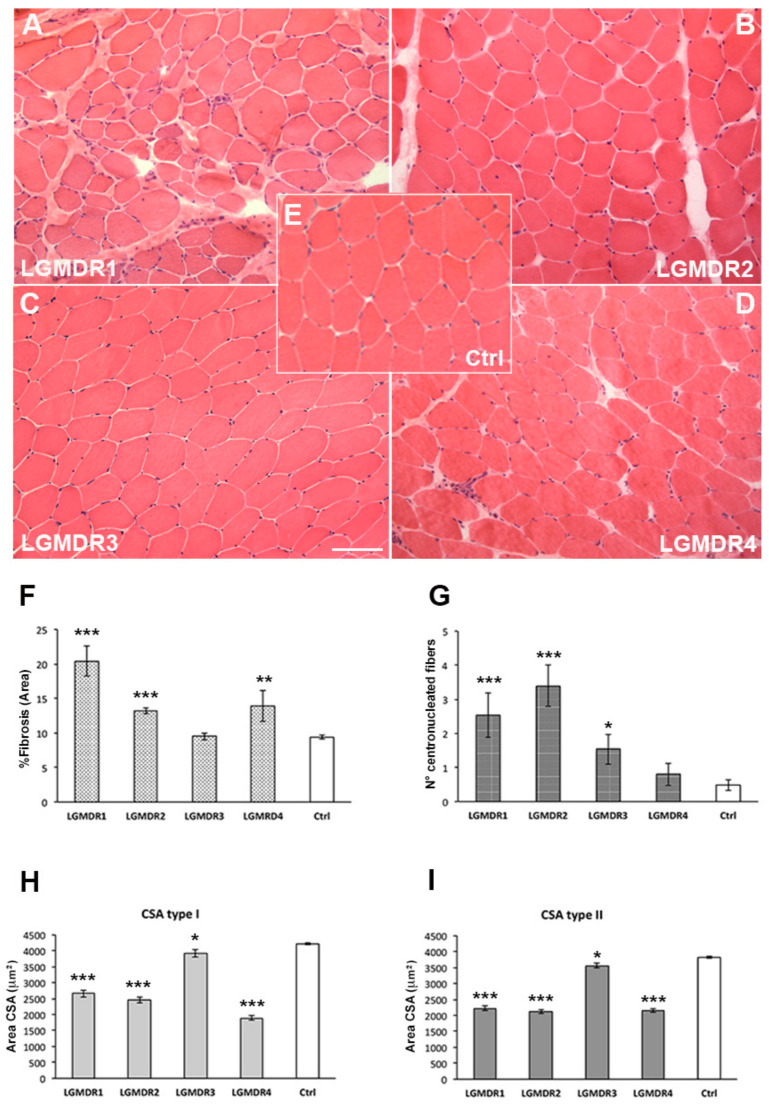
Representative images of H and E showing a variable degree of fibrosis in LGMDR1 (**A**), LGMDR2 (**B**), LGMDR3 (**C**) and LGMDR4 (**D**) patients compared to age-matched healthy controls (**E**). Scale bar 50 µm. (**F**) Fibrosis in LGMD patients and in controls. (**G**) Number of centronucleated fibers in LGMDR patients and controls, CSA of type I (**H**) and type II (**I**) fibers in LGMDR patients and controls. *p* values ≤ 0.001 (***), *p* ≤ 0.01 (**) and *p* ≤ 0.05 (*).

A histological evaluation of muscle biopsies showed a variable amount of connective tissue: a significant increase was detected in LGMDR1 (20.44 ± 2.23, *p* < 0.0001), in LGMDR2 (13.24 ± 0.45, *p* < 0.0001) and in LGMDR4 (13.90 ± 2.22; *p* = 0.0002) subjects versus age-matched controls (9.39 ± 0.29), whereas no significant changes were observed in LGMDR3 (9.49 ± 0.48; *p* = 0.8619) (Figure 1A).

A significant increase in centronucleated fibers was detected in LGMDR1 (2.53 ± 0.66; *p* < 0.0001), LGMDR2 (3.40 ± 0.61; *p* < 0.0001) and LGMDR3 (1.53 ± 0.43; *p* = 0.0081) specimens compared to age-matched controls (0.48 ± 0.16). No significant differences were observed in LGMDR4 samples (0.80 ± 1.03; *p* = 0.320) (Figure 1B).

The CSA quantification showed a significant decrease in both type I and type II fibers in all evaluated muscle sections. In detail, the CSA of type I fibers was 2657 µm^2^ ± 116 µm^2^ in LGMDR1 (*p* < 0.0001), 2451 µm^2^ ± 89 µm^2^ in LGMDR2 (*p* < 0.0001), 3920 µm^2^ ± 114 µm^2^ in LGMDR3 (*p* = 0.0145) and 1893 µm^2^ ± 67 µm^2^ in LGMDR4 (*p* < 0.0001) specimens compared to age-matched controls (4215 µm^2^ ± 38 µm^2^) (Figure 1C).

Type II fibers’ CSA was 2227 µm^2^ ± 72 µm^2^ in LGMDR1 (*p* < 0.0001), 2118 µm^2^ ± 69 µm^2^ in LGMDR2 (*p* < 0.0001), 3563 µm^2^ ± 80 µm^2^ in LGMDR3 (*p* = 0.0019) and 2158 µm^2^ ± 58 µm^2^ in LGMDR4 (*p* < 0.0001) specimens compared to age-matched controls (3819 µm^2^ ± 36 µm^2^) (Figure 1D).

### 2.2. Data Control Quality

**Figure 2:**
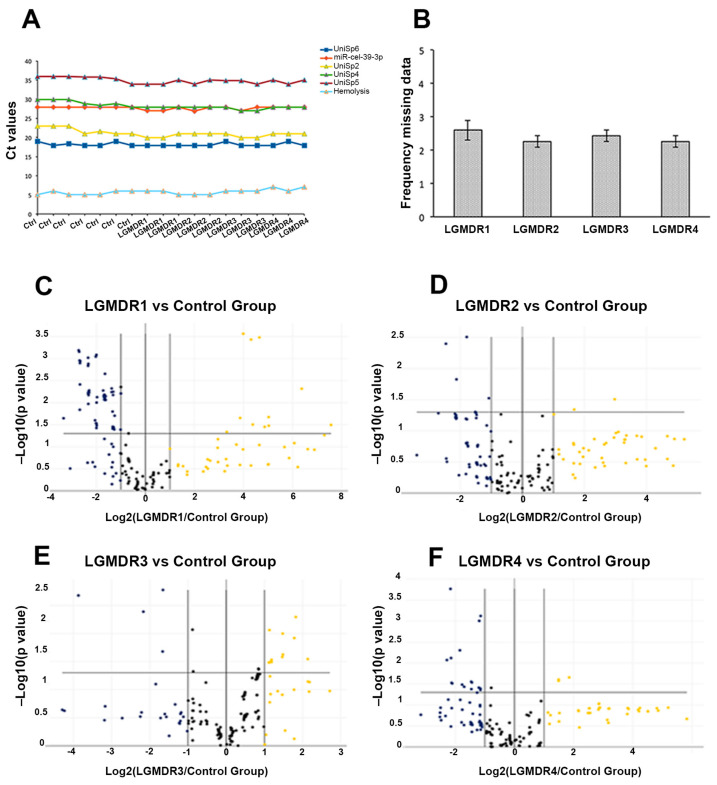
(**A**) Data quality control for efficiency of RNA isolation (UniSp2, UniSp4 and UniSp5), RT and PCR (UniSp6 and cel-miR-39-3p) and hemolysis (miR-23a-3p-miR-451a). (**B**) Frequency of missing data for each pool during RT-PCR on microarray. (**C**–**F**) Volcano plot for each LGMD pool shows distribution of miRNAs.

The Ct value results for erythrocyte contamination (miR-451a and miR-23a-3p), efficiency in RNA isolation (UniSp2, UniSp4 and UniSp5), RT and PCR inhibitors (UniSp6 and cel-miR-39-3p) in each LGMD and control sample are shown in Figure 2A.

The evaluation of the percentage of miRNAs per sample that do not reach the Ct target by the Gene Quality Plot showed no significant differences among the analyzed samples. The frequency of missing data was 2.73 ± 0.30 in the control pool, 2.60 ± 0.52 in LGMDR1, 2.25 ± 0.30 in LGMDR2, 2.42 ± 0.30 in LGMDR3 and 2.07± 0.01 in LGMDR4 (Figure 2B). MiRNA expression profiles in each patient pool are visualized in volcano plots (Figure 2C–F).

### 2.3. The Expression Analysis of Serum-Circulating miRNAs

We screened 179 miRNAs in the sera of LGMD patients and healthy controls. Patients were divided in four pools (LGMDR1-R2-R3 and R4) and miRNA analyses were performed using GeneGlobe Data Analysis Center software (https://geneglobe.qiagen.com/us/analyze; accessed on July 2022). A total of 107 miRNAs were found to be dysregulated in LGMD patients when compared to healthy controls.

**Figure 3:**
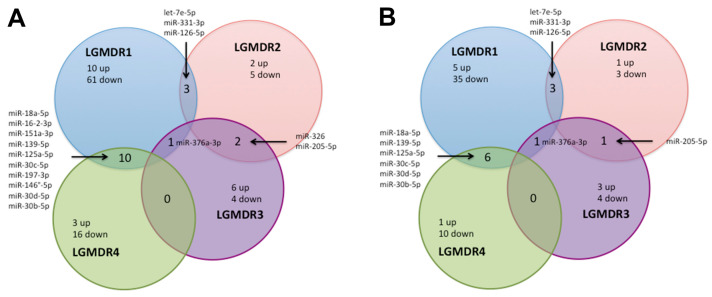
Venn graph of altered miRNAs before (**A**) and after (**B**) TSI filtering in LGMDR pools.

The most extensive number of dysregulated miRNAs was found in LGMDR1 (71), followed by LGMDR4 (19), LGMDR3 (10) and LGMDR2 (7), as depicted in volcano plots (Figure 3A). LGMDR1 and LGMDR2 shared three miRNAs (let7e-5p, miR-331-3p and miR-126-5p), LGMDR1 and LGMDR3 only shared one miRNA (miR-376a-3p), LGMDR1 and LGMDR4 had ten miRNAs in common (miR-18a-5p, miR-16-2-3p, miR-151a-3p, miR-139-5p, miR-125a-5p, miR-30c-5p, miR-197-3p, miR-146a-5p, miR-30d-5p and miR-30b-5p) and LGMDR2 and LGMDR3 had two miRNAs in common (miR-326 and miR-205-5p), as depicted in the Venn diagram (Figure 3A). The established criteria for the selection of candidate miRNAs were high expression levels (Ct values), statistical significance (*p* values) and high expression in skeletal muscle tissue (TSI values). After the application of TSI filtering, the number of dysregulated miRNAs decreased to 40 for LGMDR1, 4 for LGMDR2, 7 for LGMDR3 and 11 for LGMDR4. The number of common miRNAs did not change between LGMDR1 and LGMDR2 or between LGMDR1 and LGMDR3, whereas it decreased to five between LGMDR1 and LGMDR4 (miR-18a-5p, miR-139-5p, miR-125a-5p, miR-30c-5p and miR-30b-5p) and to one between LGMDR2 and LGMDR3 (miR-205-5p) (Figure 3B).

### 2.4. The Identification of miRNAs Differentially Expressed in Each LGMDR Pool and the Functional Enrichment of miRNA Targets

In order to identify a more specific signature for LGMD patients, the study was focused on miRNAs differentially expressed in healthy controls, filtered for their degree of expression in skeletal muscle tissue with the exclusion of those common to two or more LGMD pools.

**Figure 4:**
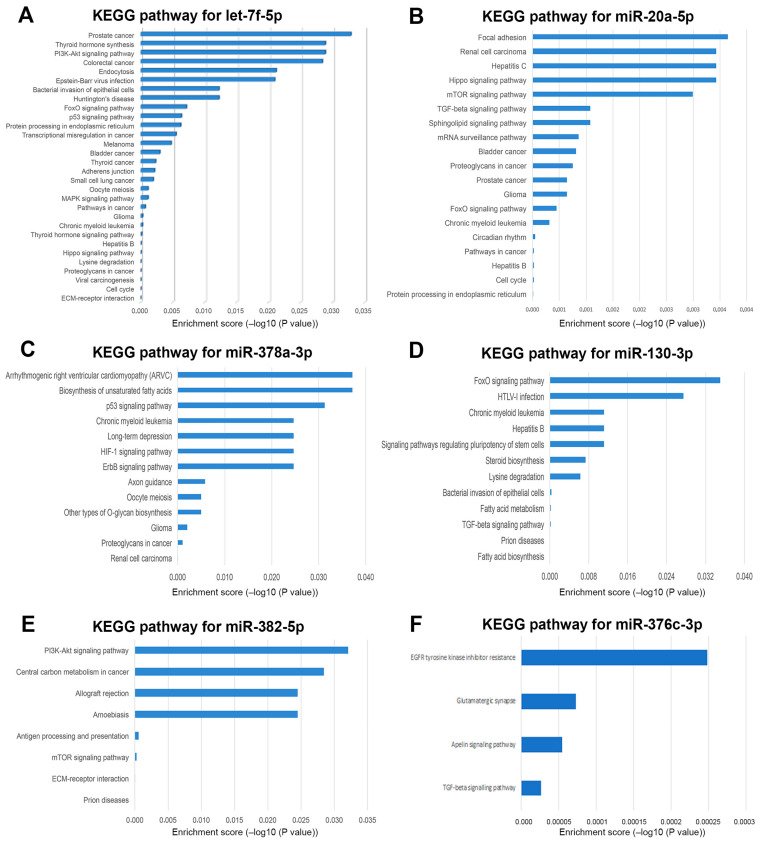
KEGG pathway analysis of let7f-5p (**A**), miR-20a-5p (**B**), miR-378a-3p (**C**), miR-130-3p (**D**), miR-382-5p (**E**) and miR-376c-3p (**F**).

**Figure 5:**
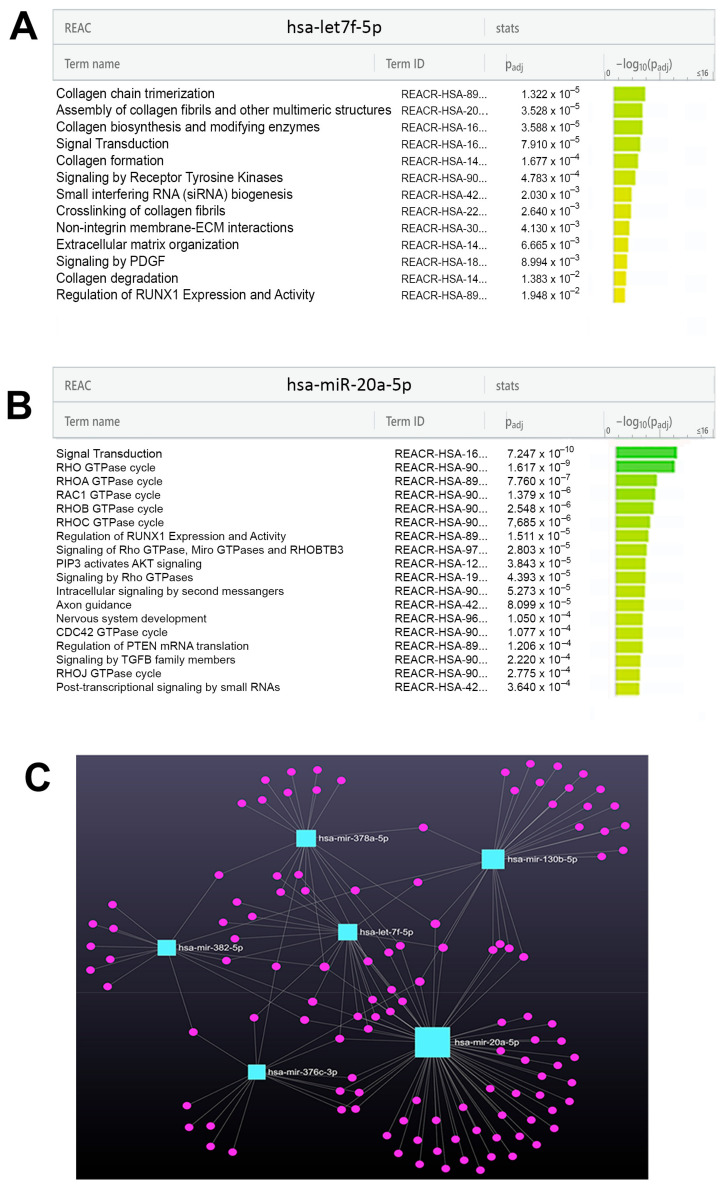
Reactome analysis on g:Profiler database for let-7f-5p showed an enrichment of pathways involved in collagen homeostasis (**A**). Reactome analysis on g:Profiler database for miR-20a-5p showed an enrichment of RHO pathway (**B**). MiRNet-target gene network for the six selected miRNAs (**C**).

In the LGMDR1 pool, a consistent number of down-regulated miRNAs belonged to the let-7 family, on which we have focused more closely. With regard to non-coding RNAs, the let-7 miRNA family represents the first known human miRNA family, consisting of let-7a/b/c/d/e/f/g/i and miR-98 [23]. The TargetScan database predicted 1191 target genes for the broadly conserved let-7 family. All members of this family have some identical seed sequences and variable stem-loop regions. Among the target genes of let-7 family, the *CAPN3* gene was identified. The TargetScan indicated 7-mer conserved sequences on 3′UTR on *CAPN3* gene for let-7i-5p, let-7g-5p, let-7a-5p, let-7f-5p, let-7b-5p, all found to be dysregulated in the LGMDR1 pool. Among the down-regulated let-7 family members in the LGMDR1 pool, we selected let-7f-5p, the most down-regulated, for further investigation. A KEGG analysis indicated that the ECM–receptor interaction was the most significant pathway (hsa04512) (Figure 4A). Further analysis of let-7f-5p target genes on the g:Profiler Reactome database revealed an interesting link with the homeostasis of collagens and ECM organization (Figure 5A). The other altered miRNAs found in LGMDR1 pool were investigated individually with the TargetScan database to identify their possible involvement in delineating the pathological traits of LGMDR1. Among the most significant recurrent pathways associated with these dysregulated miRNAs, and with possible effect on skeletal muscle, we identified the ECM–receptor interaction (hsa04512), fatty acid biosynthesis (hsa00061) and metabolism (hsa01212), TGF-beta signaling (hsa04350), the adherens junction (hsa04520), the Hippo signaling pathway (hsa04390) and proteoglycans in cancer (hsa05205).

Analysis performed on the LGMDR2 pool identified seven differently expressed miRNAs, of which two were over-expressed and five were down-expressed, compared to control pools. The two significantly up-regulated miRNAs were miR-326 and miR-205-5p, whereas the five down-regulated ones, from the highest to the lowest, were: miR-126-5p, miR-331-3p, let-7e-5p, miR-20a-5p and miR-223-5p. After TSI filtering, the remaining miRNAs were the up-regulated miR-205-5p (in common with LGMDR3) and the down-regulated let-7e-5p, miR-331-3p (in common with LGMDR1) and miR-20a-5p. A KEGG analysis on miR-20a-5p identified the protein processing in the endoplasmic reticulum (hsa04141) as the most significative pathway (Figure 4B). Further analysis of miR-20a-5p target genes on the g:Profiler Reactome database evidenced an interesting link with Rho GTPases signaling that has been linked to membrane dynamics (Figure 5B).

The analysis of the LGMDR3 pool identified ten differently expressed miRNAs; among them, six were over-expressed and four were down-expressed compared to the control pool. Of these, miR-486-5p, miR-451a, miR-376a-3p were in common with LGMDR1, and miR-133a-3p and miR-205-5p were in common with LGMDR2, whereas miR-378a-3p and miR-130b-3p, which scored a high TSI, turned out to be altered only in LGMDR3 and, therefore, they were further investigated. A KEGG analysis for miR-378a-3p showed pathways not specifically involved in skeletal muscle function except for the HIF-1 signaling pathway, reportedly involved in the development of skeletal muscle fibrosis (Figure 4C) [24]. MiR-378a-3p was found to be highly expressed in skeletal muscle with a TSI score of 0.89. MiR-378a influences vascularization in skeletal muscle [25] and promotes the differentiation of myoblasts targeting HDAC4 during skeletal muscle development [26]. MiR-378 is also associated with the activation of anti-inflammatory M2-macropahges and with the regulation of genes involved in PI3K/Akt signaling pathway [27].

A KEGG analysis of miR130b-3p, the other selectively altered miRNA in the LGMDR3 pool, found it was involved in two significant pathways, fatty acid biosynthesis (hsa00061) and TGF-beta signaling (hsa04350) (Figure 4D).

The LGMDR4 pool analysis identified three over-expressed and sixteen down-expressed miRNAs. Among the three over-expressed miRNAs, only miR-18a-5p had a TSI mean value of 0.78 and was found to be in common with LGMDR1. Among the sixteen down-expressed miRNAs, ten presented a mean TSI > 0.78. After eliminating those in common to the other groups, we deepened the study of the ones differentially expressed in LGMDR4, with miR-382-5p and miR-376c-3p being particularly interesting. The target genes prediction of the TargetScan showed a relatively limited number of target genes for both miR-382-5p (216 genes) and miR-376c-3p (266 genes). The KEGG analysis of miR-382-5p pointed out the significance of the ECM–receptor interaction (hsa04512) and the mTOR signaling pathways (hsa04150) (Figure 4E). As for the other LGMDR4 down-regulated miRNA, miR-376c-3p, the KEGG analysis targeted the TGF-beta (hsa04350) and the Apelin (hsa04371) signaling pathways (Figure 4F).

After TSI filtering, 11 miRNAs were found to be in common with all four LGMD pools (Figure 3B). The most significant among the first ten pathways revealed by their KEGG analysis were that of protein processing in the endoplasmic reticulum, the ubiquitin-mediated proteolysis, proteoglycans in cancer, the FoxO signaling pathway and the focal adhesion.

The miRNet 2.0 database identified the miRNA target genes network for the six selected miRNAs (Figure 5C).

### 2.5. Validation and Specificity Evaluation of miRNA Signature Identification in LGMD Patients

**Figure 6:**
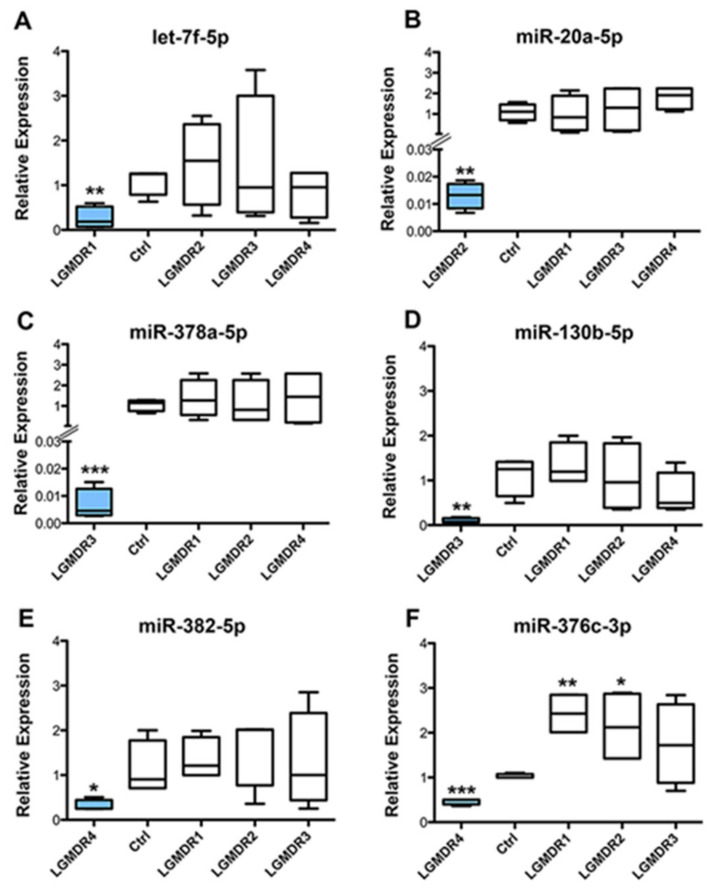
Quantitative real-time PCR validations of six differentially expressed miRNAs in LGMD single patients and controls. The relative expression of miRNAs was normalized to miR-16-5p by using the 2^−ΔΔCt^ method. let-7f-5p (**A**), miR-20a-5p (**B**), miR-378a-5p (**C**), miR-130b-5p (**D**), miR-382-5p (**E**), miR-376c-3p (**F**). Asterisks express the significance levels for each miRNA compared to control values. *p* values ≤ 0.001 (***), *p* ≤ 0.01 (**) and *p* ≤ 0.05 (*). Blue boxes indicate the relative expression value in reference patients’ pools.

The six miRNAs identified in the patients’ pool by previous analyses (let-7f-5p, miR-20a-5p, miR-378a-3p, miR-130b-3p, miR-382-5p and miR-376c-3p) were validated by RT-qPCR using individual miRNA-specific primers in single LGMD patients and controls. The results showed significantly low expression levels of the six selected miRNAs compared to controls in accordance with the data obtained from microarray analysis. The data are reported in the boxplots of Figure 6. All six miRNA expression levels in single patients confirmed the low levels found in the specific LGMD pool from microarray analysis (the blue box in each graph). The expression levels of the six miRNAs in the other LGMD patients were not significantly different compared to the controls, the only exception being miR-376c-3p in LGMDR1 (*p* = 0.0012) and LGMDR2 (*p* = 0.0351) patients (Figure 6F).

### 2.6. The Correlation of Circulating miRNAs with Demographic, Morphological and Functional Parameters

**Figure 7:**
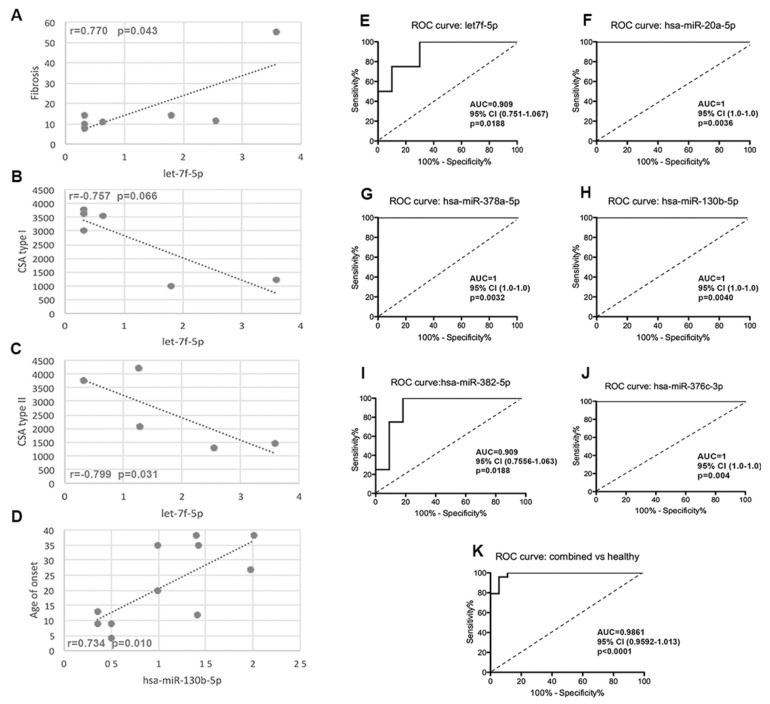
Correlation between fibrosis (**A**), CSA type I (**B**), CSA type II (**C**) and the expression values of let-7f-5p. Correlation between age of onset and the expression values of has-miR-130b-5p (**D**). (**E**–**K**) ROC analysis for each selected miRNA to distinguish among different LGMD patients: ROC analysis of let-7f-5p (LGMDR1) (**E**), miR-20a-5p (LGMDR2) (**F**), miR-378a-5p (**G**)/miR-130b-5p (**H**) (LGMDR3) and miR-382-5p (**I**)/miR-376c-3p (**J**) (LGMDR4) patients versus other LGMD patients. (**K**): ROC analysis of six-miRNAs to distinguish patients from healthy subjects.

Statistical analysis on selected miRNAs showed a significant correlation of let-7f-5p with fibrosis (r = 0.770; *p* = 0.043) (Figure 7A), CSA type I (r = −0.757; *p* = 0.066) (Figure 7B) and CSA type II (r = −0.799; *p* = 0.031) (Figure 7C). MiR-130b-5p showed a significant positive correlation with the disease age of onset (r = 0.734; *p* = 0.010) (Figure 7D).

### 2.7. The Specificity of the miRNA Signature

Since the aim of this study was to identify a possible miRNA signature to discriminate among different LGMD patients, the discriminatory value of each selected miRNA was evaluated by the ROC curves analysis. The AUCs were 0.909 (95% CI: 0.751–1.067) for let-7f-5p (Figure 7E) and for miR-382-5p (95% CI: 0.755–1.063) (Figure 7I), and 1 (95% CI: 1.0–1.0) for miR-20a-5p (Figure 7F), mir-378a-5p (Figure 7G), miR-130b-5p (Figure 7H) and miR-376c-3p (Figure 7J).

We also analyzed the diagnostic value of the six combined miRNAs as a panel to distinguish between LGMD patients and healthy controls. The AUC was 0.986 (95% CI: 0.959–1.013) (Figure 7K).

## 3. Discussion

This study was conducted to search for a miRNA signature identifying LGMD patients, the purpose being to improve patient stratification and management and to discuss the potential role of miRNAs as candidate disease biomarkers.

We identified six miRNAs—let-7f-5p (in LGMDR1), miR-20a-5p (in LGMDR2), miR-130b-5p, miR-378a-5p (both in LGMDR3), miR-376c-3p and miR-382-5p (both in LGMDR4)—whose expressions were significantly lower compared to the controls in the different LGMD pools.

We focused our attention on selected serum miRNAs whose expression in four different LGMD forms (R1-R4) was different compared to healthy controls.

In LGMDR1 patients, we focused our attention on let-7f as a negative regulator of profibrotic processes in several disease states. Let-7 has been shown to modulate ECM deposition in breast, pancreas, and oral cancer cells [28]. In addition, a decrease in let-7 was found in lung tissue samples from idiopathic pulmonary fibrosis patients [29], in renal fibrosis [30,31], and in pancreatic cancer-related fibrosis [32]. A low expression of let-7 was associated with myofibroblast differentiation and ECM deposition in human bronchial epithelial cells [33]. Two members of the let-7 family, let-7e-5p and miR-98–5p, belonging to the so called “MechanomiR”, were found to be highly dysregulated in the diaphragm of the *mdm* mouse model of muscular dystrophy [34]. In this study, the authors identified some target genes of let-7 family, such as *Col1a1*, *Col1a2*, *Col3a1*, *Col24a1*, *Col27a1*, *Itga1*, *Itga4*, *Scd1*, and *Thbs1*, demonstrating that the dysregulation of let-7 could trigger muscle fibrosis in mice models and in myoblast cultures.

A functional and physiological ECM was the result of a balance between synthesis and degradation processes. Fibrosis, the most conspicuous pathological change in muscle, is a complex, not fully understood, process characterized by the excessive accumulation of collagen and other ECM components; it is regulated by mechanisms involving cell–cell and cell–matrix interactions, as well as by factors secreted into the ECM. The ECM plays an important role in both cell signaling and homeostasis; furthermore, by impeding fiber regeneration, fibrosis may hinder the targeting of specific therapies (drugs or cells) for muscles and worsen disease progression by obstructing nutrient delivery and severely limiting patient biomechanics [35]. Our data on fibrosis quantification in LGMDR1 muscles show the highest degree of fibrosis compared to both the other LGMDR forms and the age-matched controls. These observations suggest a link between the altered expressions of different let-7 family members and the fibrotic process in LGMDR1. Among the components of the let-7 family, we further analyzed let-7f-5p, whose KEGG analysis reported the ECM–receptor interaction as the most significant pathway, whereas the analysis on Reactome identified some pathways involved in collagen assembly and in ECM organization. Our statistical analysis shows a significant correlation of let-7f-5p with fibrosis in LGMDR1 patients. The low expression of let-7f-5p could contribute to alter the ECM homeostasis resulting in fibrosis, suggesting that this miRNA could be investigated as a biomarker to monitor the progression of this pathological feature.

In LGMDR2 patients, miR-20a-5p is particularly interesting. The down-regulated miR-20a-5p and the axis miR-20a-5p/TGFBR2 are involved in inflammation during liver fibrosis. It was demonstrated that the down-regulation of miR-20a-5p in liver fibrosis resulted in TGFBR2-activated TGF-β/SMAD signaling pathway, followed by the activation of macrophage and increase in ECM deposition [36]. The KEGG analysis of miR-20a-5p indicated that the protein processing in endoplasmic reticulum was the most significant target pathway, whereas the Reactome analysis showed the important involvement of Rho signaling with a higher enrichment for Rho GTPases. Rho GTPases regulate a wide variety of cellular process, including skeletal muscle development and regeneration. Furthermore, Rho GTPases are involved in actin-mediated membrane vesicle trafficking. Dysferlin is implicated in various cellular functions, i.e., muscle cell–cell fusion during regeneration, muscle growth and cell adhesion and in plasma membrane repair in both cardiac and skeletal muscle. Skeletal muscle cells derived from LGMDR2 patients present a defective plasmalemma repair: when dysferlin expression is altered, vesicles tend to accumulate beneath the plasmalemma [37]. A critical element for vesicle trafficking and membrane repair is the actin cytoskeleton. The low expression of miR-20a-5p could contribute to the activation of the Rho GTPase pathway, ultimately worsening the vesicle membrane trafficking in LGMDR2 patients. There is an interesting link between the TGF-beta and Rho pathway as recently demonstrated in lung injury [38]. The altered expression of miR-20a-5p in LGMDR2 patients could increase the expression of the cellular components of the TGF-beta and Rho GTPase pathways, thus altering the cytoskeletal remodeling and further compromising the membrane repair mechanism. All these observations point to miR-20a-5p as a sensible miRNA biomarker for LGMDR2 pathology and progression. No significant correlations between miR-20a-5p and clinical data were detected.

Skeletal muscle in LGMDR3 patients is characterized by fiber necrosis, regeneration and inflammatory infiltrates. MiR-378a-3p was reported as being highly expressed in skeletal muscle where it influenced vascularization via the HIF-1 signaling pathway [25], which we identified by KEGG analysis, and it was found to promote the differentiation of myoblasts targeting HDAC4 during skeletal muscle development [26]. Interestingly, miR-378-3p was associated with the activation program of anti-inflammatory M2-macropahges [27]. The study also identified several targets for miR-378a-3p within the PI3K/Akt signaling pathway, which are important for proliferation, but only partially responsible for the M2 phenotype [27]. In LGMDR3 patients, another interesting miRNA, miR-130b-3p, was identified. This miRNA was associated with the molecular regulation of macrophages’ polarization by IRF1 as a target gene. An overexpression of miR-130b-3p alleviates the inflammation of lung tissue by suppressing M1 polarization in a murine model treated with LPS [39]. The KEGG analysis indicated its involvement also in the fatty acid biosynthesis and in the TGF-beta signaling pathways. We hypothesize that a combined action of dysregulated miR-378a-3p and miR-130b-3p could play a role in the inflammatory response and, in particular, in the polarization of macrophages in LGMDR3 patients.

In LGMDR4 patients, the main focus was on miR-382-5p and miR-376c-3p, with the former reportedly involved in mitochondrial dynamics. In an in vitro model of murine myotubes, miRNA-328-5p silencing resulted in an induction of several genes involved in mitochondrial dynamics (MFN-1, MFN-2 and OPA-1) and biogenesis (SIRT1 and PGC-1α) and in the activation of the mitochondrial unfolded protein response [40]. Furthermore, miR-382-5p was associated with mitochondrial pathways related to different neuromuscular disorders [41]. Another interesting miRNA, down-regulated in LGMDR4, is miR-376c-3p. A target gene of the conserved miR-376c-3p is the muscle atrophy gene-1 (Atrogin-1). Shin and colleagues showed that the intramuscular delivery of AAV9-expressing miR-376c-3p ameliorated skeletal muscle atrophy and improved muscle function in old mice and proposed that miR-376c-3p would be a valuable candidate for the development of therapies aimed at maintaining muscle homeostasis during aging [42]. The marked reduction in CSA for both type I and type II fibers observed in LGMDR4 patients suggested a condition of muscle atrophy. Muscle atrophy could result from a disorder in protein synthesis and catabolism with the involvement of different pathways such as the ubiquitin proteasome system (UPS), the autophagy pathway and the caspase system. The Reactome analysis of miR-376c-3p showed different interactions with the IGF1R signal cascade and with IRS-related events triggered by IGFR1R. IGF1 plays a role in skeletal myogenesis and is importantly associated with muscle mass entity and strength development; also, it increases the proliferative capacity of muscle satellite cells [43]. Recently, a circulating miRNome analysis was performed on six LGMD serum patients (three *CAPN3-*, two *TTN-* and one *SGCA*-mutated patients) to identify a molecular miRNA signature for patient prognosis and stratification. Among the different expressed miRNAs, the authors identified a combination of miR-122-5p, miR-192-5p and miR-323-3 as potential molecular miRNA signatures for LGMD patient prognosis even if the data need to be confirmed in a larger cohort of patients [44].

The evaluation of ROC curves showed that the selected miRNAs were able to discriminate among different LGMD patients as the combined six-miRNAs were able to discriminate between LGMD patients and controls.

### Study Limitation

The sample size in the study was small, but only relatively so if we consider the rarity of these diseases. We are planning to develop this project by a further step by trying to involve other rare disease centers to increase the sample size. Also, we mean to recruit any other subjects who may address our hospital for the first time.

## 4. Materials and Methods

### 4.1. Patients

Sixteen LGMD patients affected with LGMDR1, LGMDR2, LGMDR3 and LGMDR4 were analyzed. For preliminary screening, the patients’ sera were grouped in four pools. Two pools of age-matched control sera were also analyzed. The demographic, clinical and functional characteristics of LGMD patients and healthy controls involved in study are summarized in Table 1.

### 4.2. Ethics Statement

All procedures were conducted in accordance with the standards of the local Ethics Committee and the Declaration of Helsinki. All involved subjects had signed their written informed consent before undergoing blood sampling and/or skeletal muscle biopsy.

### 4.3. Morphology

Muscle biopsy was available for some of the patients. Routine Hematoxylin and Eosin (H and E) histology was performed on 8 μm thick cross cryostat muscle sections and the fibrotic area (connective and adipose tissue) was quantified using Leica Application Suite 4.9.0 and ImageJ 1.53c (https://imagej.nih.gov/ij/download.html; accessed on February 2022) software. ATPase pH 9.6 staining was also performed on muscle sections to evaluate cross sectional area of type I and type II fibers [45]. For both analyses, on each section, four randomly, non-overlapping, selected fields were photographed at 20× magnification, using optical microscope Leica DC200 equipped with a camera and IM50 image analysis software Leica Application Suite v4 (Leica Microsystems, Wetzlar, Germany).

### 4.4. Serum Collection

Peripheral venous blood was collected in serum collection tubes (Greiner Vacuette Serum Separator Tubes, 456079). To isolate serum, blood was centrifuged at 1800× *g* for 12 min at room temperature. Supernatant was collected and stored frozen in aliquots at −20 °C pending use.

### 4.5. RNA Isolation

Serum was thawed in ice, rapidly vortexed and centrifuged at 1000× *g* for 5 min in a microfuge. Total RNA, including miRNA fraction, was extracted from 200 µL of serum using the Qiagen miRNeasy Serum/Plasma kit according to manufacturer’s instruction. RNA fraction was finally eluted in 20 µL of nuclease-free water and stored at −80 °C. An RNA spike-in mix (UniSp-2, UniSp4 and UniSp5; Qiagen, Hilden, Germany) at three different concentrations was added to lysis buffer during RNA isolation to monitor RNA extraction efficacy. Both quality and quantity of RNA were evaluated by 260/280 nm ratio using NanoDrop spectrophotometry (NanoDrop Technologies, Wilmington, DE, USA).

### 4.6. cDNA Synthesis

RNA was reverse transcribed using miRCURY Locked Nucleic Acid (LNA™) Universal Reverse Transcription (RT) kit (Qiagen) according to manufacturer’s instruction. In brief, 4 µL of RNA was reverse transcribed in a final volume of 20 µL. The reaction mix was incubated at 42 °C for 1 h and then inactivated at 95 °C for 5 min. RT sample was stored at −20 °C until use. The cDNA synthesis control (UniSp6) (Qiagen) and the cel-miR-39-3p spike-in (Qiagen) were added in reverse transcription reaction to evaluate the efficacy of RT reaction.

### 4.7. RT-PCR Based miRNA Assays (Real Time PCR)

The Serum/Plasma miRNA Focus PCR Panel comprising a set of two 96-well plates was used for the analysis of 179 miRNAs. cDNA samples were amplified using miRCURY LNA SYBR Green PCR kit (Qiagen); RT-PCR was performed in ABI 7500 Real Time PCR System (Applied Biosystems, Foster City, CA, USA) for both Ct detection and melting curve analysis. RT-PCR conditions were: 1 cycle of heat inactivation at 95 °C for 2 min, 40 cycle of amplification for 10 s at 95 °C (denaturation) and 60 s at 56 °C (annealing/extension). RT-PCR data were analyzed using GeneGlobe Data Analysis software (http://www.qiagen.com/genglobe; accessed on March 2022) (Qiagen) in which the RT-PCR modules converted the threshold cycle (Ct) values into the calculated results for miRNA expression and also via the comparative Ct method using 2^−ΔΔCt^ [46]. Threshold cycle (Ct) values >35 were excluded. Fold change cut-off was ≥2.0 for up-regulated genes and ≤0.5 for down-regulated genes. The miRNA expression profile was normalized by global Ct mean of expressed miRNAs according to GeneGlobe Data Analysis-processed data. The *p* values were calculated based on a Student’s *t*-test. The *p* value calculation used is based on parametric, unpaired, two-sample equal variance and two-tailed distribution (as reported by Qiagen).

### 4.8. Real-Time Quantitative PCR (RT-qPCR)

To confirm the data obtained by miRNA array, the expression of each miRNA was measured using miRCURY LNA miRNA detection probes (Qiagen) and SYBR Green PCR method. The single miRNAs analyzed were let-7f-5p (YP00204359), miR-20a-5p (YP00204292), miR-378a-3p (YP00205946), miR-130b-2p (YP00204317), miR-376c-3p (YP00204442) and miR-382-5p (YP00204169). Each sample was analyzed in duplicate and values were averaged. Serum miRNAs were normalized using miR-16-5p as endogenous control and applied the Ct method 2^−ΔΔCt^. Analyses of these miRNAs were performed on serum of each pooled patient and on eight further LGMD patients.

### 4.9. Data Control Quality

Among 179 miRNAs tested in Serum/Plasma miRNA-focused PCR Panel, some were used as quality controls. Serum hemolysis was analyzed with miR-23a-3p and miR-451a. MiR-23a-3p was stably present in serum/plasma and was not affected by hemolysis, while miR-451a was expressed in red blood cells. The ratio between these two miRNAs correlated with the hemolysis degree; samples with a ratio above 7 were considered affected by hemolysis and excluded. UniSp2, UniSp4, UniSp5, UniSp6 and cel-miR-39-3p spike-in controls added during RNA extraction and reverse transcription reaction were analyzed according to GeneGlobe Data Analysis-processed data. Interplate calibrator (IPC-UniSp3) was used for comparing data for some samples analyzed on multiple plates requiring different runs according to GeneGlobe Data Analysis-processed data.

Data quality also included the evaluation of percentage of miRNAs per sample that did not reach the Ct target by the Gene Quality Plot.

### 4.10. Bioinformatics Tools

The miRNA expression in human skeletal muscle tissues was checked by Tissue Atlas database (https://ccb-web.cs.uni-saarland.de/tissueatlas/; accessed on August 2022). Tissue specificity index (TSI) values ranged from less than 0.5 for housekeeping miRNAs to more than 0.85 for tissue-specific expression. TSI was verified for each miRNA analyzed [47]. The TSI cutoff for skeletal muscle expression applied was ≥0.7. TargetScan (http://targetscan.org/vert_71/; accessed on May 2022) database was used for gene target prediction. Kyoto Encyclopedia of Gene and Genome (KEGG) pathway enrichment and Gene Ontology (GO) analysis were performed with mirPath v.4 (https://diana-lab.e-ce.uth.gr/app/miRPathv4; accessed on July 2022). g:Profiler (http://biit.cs.ut.ee/gprofiler/gost; accessed on September 2022) [48] and MiRNet (https://mirnet.ca/miRNet/home.xhtml; accessed on September 2022) were consulted for Reactome analysis.

### 4.11. Statistical Analysis

Statistical analysis was performed using GraphPad Prism 5 (GraphPad Software Inc., LaJolla, CA, USA). Data of functional tests are expressed as mean ± SD; morphological data are expressed as mean ± SEM. Data were tested for normal distribution using ANOVA test. Two tails unpaired *t*-test with confidence interval of 95% was applied to analyze data. Significance levels were set as *p* ≤ 0.001 (***), *p* ≤ 0.01 (**) and *p* ≤ 0.05 (*).

Spearman’s correlation analyses were applied to assess correlation between miRNA levels and clinical, histological and molecular characteristics of patients. Difference between patients and controls was assessed with Wilcoxon-Mann–Whitney test. Receiver Operating Characteristics (ROC) curves to define the diagnostic performance of miRNAs were plotted to determine the area under the curve (AUC).

## 5. Conclusions

To identify a specific signature of circulating miRNAs in LGMD patients, a microarray of 179 serum miRNAs, not including myomiRs, were analyzed for their serum expression in a cohort of LGMD patients. From this study, we identified six differently expressed miRNAs that can be evaluated as possible biomarkers for LGMD patients and as possible therapeutical targets, even if a larger sample size is needed to replicate and validate these data. Though it is true that the number of available samples did not allow us to obtain a more robust statistical analysis, it is worth considering that we are dealing with rare diseases, which largely affects the possibility of obtaining samples. Considering all this, even if a confirmative study on in vitro models is required, we believe our data strongly suggest the possible role of these selected miRNAs as non-invasive biomarkers to monitor disease progression and as targets for eligible therapeutic approaches.

## Figures and Tables

**Table 1:** Demographic, clinical and functional features of participants in the study.

Patients	LGMDR1	LGMDR2	LGMDR3	LGMDR4	Ctrl
	(N = 4)	(N = 4)	(N = 4)	(N = 4)	(N = 8)
Sex	1F/3M	3F/1M	3F/1M	1F/3M	4F/4M
Age	47.25 ± 15.51	38.25 ± 14.52	46.75 ± 16.25	19.50 ± 6.61	37.70 ± 0.63
Age at onset (yrs)	30.75 ± 7.88	20.75 ± 12.33	12.50 ± 5.44	7.25 ± 4.03	NA
Disease duration (yrs)	16.5 ± 10.37	17.25 ± 5.85	32.50 ± 17.74	9.0 ± 8.36	NA
**Morphology**					
Fibrosis (% area)	20.44 ± 2.23 *p* < 0.0001	13.24 ± 0.45 *p* < 0.0001	9.49 ± 0.48 *p* = 0.8619	13.90 ± 2.22 *p* = 0.0002	9.39 ± 0.29
Centronucleated Fiber	2.53 ± 0.66 *p* < 0.0001	3.40 ± 0.61 *p* < 0.0001	1.53 ± 0.43 *p* = 0.0081	0.80 ± 1.03 *p* = 0.320	0.48 ± 0.16
CSA type I (mm^2^)	2657 ± 116 *p* < 0.0001	2451 ± 89 *p* < 0.0001	3920 ± 114 *p* = 0.0145	1893 ± 67 *p* < 0.0001	4215 ± 38
CSA type II (mm^2^)	2227 ± 72 *p* < 0.0001	2118 ± 69 *p* < 0.0001	3563 ± 80 *p* = 0.0019	2158 ± 58 *p* < 0.0001	3819 ± 36
**Clinical data**					
CK (IU/L)	1813 ± 550	931 ± 185	1996 ± 1457	6151 ± 1958	60-190
Loss of ambulation	1/4	1/4	1/4	2/4	NA
**Functional tests**					**Normal values**
MFM Dimension 1 (score)	14.75 ± 15.79	23.25 ± 9.84	21.75 ± 16.45	17.25 ± 20.25	39
MFM Dimension 2 (score)	30.00 ± 5.16	30.00 ± 14.69	32.00 ± 6.73	19.00 ± 19.69	36
MFM Dimension 3 (score)	18.75 ± 1.79	18.25 ± 9.33	20.00 ± 1.15	13.00 ± 8.90	21
MFM tot (score)	63.50 ± 21.79	71.5 ± 30.50	73.75 ± 23.76	49.25 ± 48.50	96
Six Minute Walking test (6-MWT) (meter)	284.3 ± 141.8 (n = 3)	381.0 ± 152.0 (n = 3)	461.2 ± 168.8 (n = 3)	530.5 ± 67.1 (n = 2)	615.5 ± 31.8 (n = 102) [22]
NSAD	21.00 ± 22.68	32.25 ± 15.41	31.00 ± 22.13	23.00 ± 27.00	54
PUL	27.25 ± 10.14	35.00 ± 15.15	34.00 ± 10.09	22.50 ± 20.85	42

Morphological data are expressed as mean ± SEM; data of functional tests are expressed as mean ± SD. LGMD: Limb Girdle Muscle Dystrophy; CSA: cross sectional area; CK: creatine kinase; MFM: Motor Function Measurement; NSAD: North Star Assessment for limb girdle; PUL: Performance Upper Limb. NA: not applicable.

## Data Availability

The data presented in this study are available on reasonable request from the corresponding author.
